# Supplementary material for: Functional trait identity regulates productivity better than tree diversity and structural complexity in subtropical mixed-species forests
Source: PLoS One. 2025 May 30;20(5):e0324541. doi: 10.1371/journal.pone.0324541 (PMC12124508; doi:10.1371/journal.pone.0324541)
Supplement: Attachment — Submitted filename: Response to Reviewers.docx [file pone.0324541.s002.docx]

Dear Editor-in-Chief,

We express our gratitude to the Editor-in-Chief for considering our research article and providing us with the valuable opportunity to revise. We appreciate the guidance and insightful suggestions provided by the Editor-in-Chief and the reviewers, which have significantly contributed to improving the quality of our article.

We have meticulously addressed each suggestion made by the reviewers, making careful revisions throughout the entire manuscript. If any further clarification or modification is needed, please feel free to contact us. We are committed to ensuring that our article meets the standards of the journal.

Thank you for your thoughtful consideration. We look forward to hearing from you soon.

Yours sincerely,

Xiao Yu Chen

On behalf of all co-authors

Reviewer #1: The content of the manuscripts falls perfectly in the scope of PLOS ONE as it focuses on the main drivers of forest productivity including topographic diversity as well as species richness and functional diversity. The interest in the article is highly increased by the very robust dataset used comprising 120 plots. The statistical analyses is sound and well explained. The manuscript falls short on a few points, some of which are very relevant. Please find below specific comments.

Paragraph 2.2 Forest inventory and collection of plant functional traits
Since only two census were performed there is no need to say every 5 years. Simply say when the first was done and when the second was done. Something like: “a first tree census was performed in feb-2016 while a second one was performed in xx-xxxx”. Please also specify what was measured

RE: Thank you for your suggestion. We have explained the timing of the two surveys in the article and elaborated on the data measured during the survey. Please refer to lines 110-118 for the updated text.

The phrase “For all tree species, we collected two key indicators….” Is misleading and makes one think you measured the traits in you your experiment. Perhaps say directly that traits were retrieved from the try.db and from iplant.cn. Please also state (perhaps in a supplementary table) how many values you retrieved for each species and if and why you eliminated any before averaging. Also include the resulting standard deviation of averaging multiple values. I’m not familiar with iplant.cn but in the try-db you must put care in selecting the trait values as often some are outliers simply because they were taken in some peculiar environmental condition that is not stated in the database. Some data have ancillary information on the sample including environment (forest, urban, potted, etc), dominance (dominated, codominant, dominant), form (shrub, tree). All these abiotic and biotic conditions have an effect on traits. Note also that “Maximum Height” in the Try-DB often contains the height of the individual and should not be used “as is” to calculate the maximum height of a species. For example, if you average all the maximum heights in the Try –db for Quercus ilex, you get a value around 6m where the true maximum height of the species is around 14m. Did you filter the data and how did you filter it to avoid these potential biases?

RE: Thank you for your suggestion. Thank you very much for the reviewers' suggestions, which have significantly expanded our understanding. We would like to provide a detailed explanation.When collecting the functional traits of the two studied plant species, we were able to retrieve data on the maximum tree height for over 97% of the species in our plots from the website https://iplant.cn, with only a few rare species not found. For these rare species, we searched within the same genus and closely related species to estimate an approximate maximum tree height.Regarding wood density, one of our students measured the wood density of over 30 dominant species in our plots. For other species, we supplemented our data using the website [http://datadryad.org/handle/10255/dryad.235](https://www.try-db.org)), where we found data for most species, although about 10 species still lacked relevant information. In such cases, we substituted missing data with traits or averages from related genera. Additionally, we compared our results with numerous previous studies and found that our findings are consistent with theirs.

Paragraph 2.3

line 109-110. Although cited it is much more clear if you sate in the text that the allometric relationships were taken from “China’s Forest Ecosystem Carbon Storage-Biomass Equation”.

RE: Thank you for your suggestion. We have made changes in the article and see lines 122-124 for the updated text

Lines 118-119: I believe Stand Basal Area and standard deviation of Basal area are more appropriate to represent stand density and complexity. The way it is stated it looks as if the DBH of the biggest tree (and not the sum of the DBH of all trees) is used to represent stand density. Anyway, summing the DBH instead of Basal Area is tricky because of the quadratic relationship between area and diameter. In the discussion you speak of stand density and of large diameter trees so that I am confused of what is what. Please clarify in material and methods. If the diameter of the biggest tree is explaining productivity of the plot this should be thoroughly argued. Note that the bigger the tree the less space for other species in a 20 x 20 m plot so that this metric could be biased.

RE: Thank you for your suggestion. This issue has indeed caused us some confusion. Regarding the relevant indicators of structural diversity, complexity or structural properties, we have expressed some metrics based on previous studies that have utilized these indicators. The CV, Stdv, and DBHmax all demonstrate very good correlations. Considering the scientific questions we aim to address in our paper, we have taken these indicators into account comprehensively. See the following articles

Aponte, C., Kasel, S., Nitschke, C. R., Tanase, M. A., Vickers, H., Parker, L., Fedrigo, M., Kohout, M., Ruiz-Benito, P., Zavala, M. A., Bennett, L.T.. 2020. Structural diversity underpins carbon storage in Australian temperate forests. Glob. Ecol. Biogeogr. 29(5), 789–2824.

Fotis, A.T., Murphy, S.J., Ricart, R.D., Krishnadas, M., Whitacre, J., Wenzel, J.W., Queenborough, S.A., Comita, L.S., 2017. Aboveground biomass is driven by mass‐ratio effects and stand structural attributes in a temperate deciduous forest. J. Ecol. 106(2), 561–571.

Ouyang, S., Xiang, W., Wang, X., Xiao, W., Chen, L., Li, S., Sun, H., Deng, X., Forrester, D.I., Zeng, L., Lei, P., Lei, X., Gou, M., Peng, C., 2019. Effects of stand age, richness and density on productivity in subtropical forests in China. J. Ecol. 107(5), 2266–2277.

Wu, A., Tang, X., Li, A., Xiong, X., Liu, J., He, X., Zhang, Q., Dong, A., Chen, H., 2022. Tree diversity, structure and functional trait identity promote stand biomass along elevational gradients in subtropical forests of southern China. J. Geophys. Res-Biogeo. 127(10), e2022JG006950.

3. Results
Line 154: is this the annual productivity or the productivity over 5 years? Please specify and perhaps adjust measuring units.

RE: Thank you for your suggestion. We have changed it in the article, where it annual productivity

Line 154 “In this 120 species” change to “out of the 120 species….”
RE: Thank you for your suggestion.We have made changes in the text, see line 173 for the updated text

Line 157-158: “Stand productivity of Fagaceae is the largest (12.60Mg, 40.04%) in THIS forest PLOT” is not clear. Did you mean in THESE forest PLOTS.

RE: Thank you for your suggestion. Within a 6-ha plot, See line 177.

Lines 160-163: Among two species traits, tree maximum height was significantly and positively correlated with productivity (R^2^ = 0.22, P＜ 0.001; Figure 1d), while wood density was negatively correlated with productivity (R^2^ = 0.05, P＜0.05; Figure 1e).
You can start with “ among THE two FUNCTIONAL traits”. Note that the most used term for traits is FUNCTIONAL TRAITS and not SPECIES traits and you should amend throughout the manuscript.
You have already defined Maximum Height and Wood Density so you can use the commonly used acronyms afterwards: MH and WD.
RE: Thank you for your suggestion. I have revised it. See lines 180-182.

Fig 1. Please add more information in the captions. Also note that in fig 1a, Y-axis is labeled “proportion”: proportion of what? Also fig 1c , x-axis, is labeled “abundance” but abundance was not defined earlier. Is this number of individuals, basal area (maybe a proxy of density).
RE: Thank you for your suggestion. I have revised it. See Figure 1 and See lines 184-185.

Lines 168-169: “For taxonomic diversity indices, species richness was negatively correlated with productivity (R2=0.01, P=0.16)”. The correlation is not significant so that you cannot make this statement. You can only say they are not correlated. Same goes for the Pielou index few lines below.

RE: Thank you for your suggestion. We have made changes in the article. Please refer to lines 188-191 for the updated text

Lines 217-218: “The effects of taxonomic and functional diversity on forest productivity demonstrate that the complementarity effect is not prominent in this forest”. Caution must be taken in this statement (also revise the introduction): in order to compute the complementarity effect and in general to compute the “TRUE” diversity effect (let it be species richness or functional dispersion or Rao’s index) you need to compute the difference between the observed productivity and the expected productivity where the expected productivity in a stand composed by species A, B, and C is equal to the productivity of the species A, B, C when grown in monoculture weighted by the abundance in the mixed plot. See M Loreau, A Hector - Nature, 2001 - nature.com for a full explanation.

Since you do not have the productivity of the monocultures for your species you can only say that diversity metrics are not good predictors but you cannot rule out that positive interactions between species are not taking place. Also note that most of your plots have a Species Richness (SR) between 10 and 17 and that the minimum is around 5. Most usually, the SR effects is already saturating at around 5-8 so that you are likely to missing out the positive effects of diversity. Finally, note that Rao’s index also tends to saturate with increasing SR and that your correlation is not significant: again you cannot rule out a diversity effect. This is perhaps my major comment and the whole manuscript, including the objectives, must be revised clearly state what the analysis can answer to. Perhaps also the hypothesis should be revised accordingly.

RE: Thank you very much for the reviewer's suggestions. That’s a very good idea. We have made detailed revisions. See Lines 236-246.

Line 233: “These results support the mass-ratio hypothesis in this forest”. The mass-ratio hypothesis is very relevant here, so relevant that, together with the concepts of complementarity and selection (or identity) effect, it should be discussed in the introduction and add the relative references.
RE: Thank you very much for the reviewer's suggestions. The introduction provides a comprehensive discussion of the mass ratio effect and complementary effect, supplemented by relevant references. For the updated version, see lines34-49 and 275-279.

Lines 233 and 234: “complex complexity” please revise. It is used twice.
RE: Thank you for your suggestion. We have made some changes in the text. See lines 249.

Lines 235: “A variety of studies have demonstrated that structural diversity had a greater impact on forest productivity than taxonomic and functional diversity [18,32]”. This is true, but you should also acknowledge that structural complexity is often correlated with taxonomic diversity especially when the stands includes a c dominated layer as in this case. Indeed you SEM shows that a relevant part of structural diversity is explained by taxonomic diversity. To me this suggests that one important way in which taxonomic diversity is acting is by increasing the structural diversity which is well known to increase light capture and use at stand level.
By the way, it would be nice to include the correlation between taxonomic diversity and Rao’s index. Please also state which traits you used to compute Rao’s Index and be explicit in saying that Rao’s index was not a good predictor when accounting for those traits. I am asking so because it could have been that using other traits (shade tolerance for example) might have yielded different results. Consider using also Functional Dispersion (FDis in the same R Package you used) if you would like to account for categorical values as shade tolerance. Please also provide the correlation between WD and MH.

RE: Thank you for your suggestion. We calculated the functional divergence (FDis) index using the FD package, and we found a strong correlation with Rao’s index. Both indices have been widely used in many studies, and we believe that either index can effectively represent functional trait diversity.

Rao

| FDis | fds |
| --- | --- |
| 1.12329 | 1.52894 |
| 1.52922 | 2.96782 |
| 1.67059 | 3.52939 |
| 1.57503 | 2.98637 |
| 0.901643 | 1.02947 |
| 1.11261 | 1.57318 |
| 1.30524 | 2.04328 |
| 1.25505 | 1.96241 |
| 1.6267 | 2.89447 |
| 1.53683 | 2.47234 |
| 1.29121 | 2.0545 |
| 0.989848 | 1.34824 |
| 1.1998 | 2.30929 |
| 1.75826 | 3.63139 |
| 2.06564 | 4.65335 |
| 1.72373 | 3.46527 |
| 1.383 | 2.47547 |
| 1.65095 | 3.17524 |
| 1.52982 | 2.77314 |
| 1.04429 | 1.49518 |
| 1.03659 | 1.42862 |
| 1.11941 | 1.73985 |
| 1.12023 | 1.73199 |
| 1.58203 | 2.64271 |
| 1.55516 | 2.5589 |
| 0.824469 | 0.892002 |
| 1.44599 | 2.26795 |
| 1.69885 | 3.11389 |
| 1.41449 | 2.41645 |
| 1.79771 | 3.52866 |
| 1.90158 | 3.84198 |
| 1.24717 | 1.84397 |
| 1.21812 | 1.91161 |
| 1.45303 | 2.69793 |
| 1.1469 | 1.851 |
| 1.07038 | 1.36349 |
| 1.11973 | 1.62623 |
| 1.02193 | 1.40436 |
| 1.17014 | 1.80559 |
| 1.13686 | 1.77053 |
| 1.26525 | 1.84963 |
| 1.222 | 1.60322 |
| 1.27691 | 2.1882 |
| 1.27358 | 1.97448 |
| 1.62149 | 2.81714 |
| 1.71754 | 3.41828 |
| 1.33039 | 2.0505 |
| 1.51958 | 2.7197 |
| 1.19834 | 1.90497 |
| 1.15738 | 1.86289 |
| 0.762599 | 0.875584 |
| 0.783522 | 0.785965 |
| 0.619022 | 0.581657 |
| 0.850878 | 0.971468 |
| 0.908206 | 1.06062 |
| 0.886507 | 1.04465 |
| 1.15158 | 1.83282 |
| 1.63086 | 3.18773 |
| 1.56732 | 2.91611 |
| 1.54305 | 2.68469 |
| 1.35781 | 2.32291 |
| 1.10623 | 1.74347 |
| 1.24737 | 2.06136 |
| 0.989387 | 1.29993 |
| 0.800887 | 0.795306 |
| 0.940639 | 1.16529 |
| 0.958863 | 1.36435 |
| 0.847391 | 0.999109 |
| 0.85175 | 0.955882 |
| 0.947252 | 1.12447 |
| 1.01225 | 1.4257 |
| 1.1665 | 1.67184 |
| 1.3073 | 2.53935 |
| 1.52803 | 2.69615 |
| 1.66807 | 3.15566 |
| 1.65585 | 3.14299 |
| 1.42083 | 2.46307 |
| 1.10451 | 1.65787 |
| 1.08074 | 1.44927 |
| 0.714149 | 0.788053 |
| 1.09224 | 1.53635 |
| 0.886105 | 1.21434 |
| 1.15057 | 2.08596 |
| 1.19795 | 1.81829 |
| 1.28388 | 2.31887 |
| 1.64483 | 3.18691 |
| 1.16625 | 1.90572 |
| 1.6786 | 3.14963 |
| 1.58863 | 2.82178 |
| 1.57889 | 2.87373 |
| 1.53752 | 2.70155 |
| 1.3606 | 2.28814 |
| 1.31909 | 1.88386 |
| 1.47903 | 2.37286 |
| 1.15105 | 1.72032 |
| 0.966681 | 1.24845 |
| 1.21758 | 2.34496 |
| 1.40659 | 2.44863 |
| 1.43505 | 2.43188 |
| 1.38301 | 2.59147 |
| 1.71326 | 3.35928 |
| 1.96073 | 4.36069 |
| 1.43913 | 2.7675 |
| 1.55373 | 2.86085 |
| 1.71315 | 3.28828 |
| 1.42647 | 2.17265 |
| 1.71033 | 3.27297 |
| 1.49822 | 2.62069 |
| 1.30535 | 2.03562 |
| 1.1082 | 1.55895 |
| 1.37507 | 2.48601 |
| 1.39885 | 2.2252 |
| 1.57535 | 2.88694 |
| 1.59022 | 3.16697 |
| 1.31667 | 2.30589 |
| 1.39427 | 2.56149 |
| 1.67253 | 3.32758 |
| 1.45354 | 2.3771 |
| 1.34798 | 2.4372 |
| 1.71876 | 3.44944 |
| 1.41249 | 2.43959 |
| 1.21963 | 1.97066 |
| 1.47134 | 2.32714 |
| 1.73091 | 3.41612 |
| 1.83619 | 4.03087 |
| 1.89748 | 4.15772 |
| 1.42026 | 2.23595 |
| 0.997504 | 1.75185 |
| 1.04097 | 1.69343 |
| 1.0788 | 1.75361 |
| 1.25596 | 2.18267 |
| 1.18364 | 1.99282 |
| 1.49403 | 2.60414 |
| 1.14245 | 1.83601 |
| 1.43144 | 2.59136 |
| 1.63332 | 3.14248 |
| 1.58035 | 2.73253 |
| 1.54597 | 2.64374 |
| 1.76334 | 3.41222 |
| 1.43007 | 2.4996 |
| 1.5975 | 2.92518 |
| 1.56885 | 2.94803 |
| 1.4007 | 2.22709 |
| 1.32154 | 2.31837 |
| 1.22112 | 1.91416 |
| 1.15452 | 2.04402 |
| 1.47832 | 2.53673 |

Lines 247-248: you report the result of SEM that suggest an effect of topographic diversity (mostly slope and convexity) on taxonomic diversity. It would be nice if you could elaborate on this. Is it just a casual correlation in your case or can you support the occurrence of a causal relationship.

RE: Thank you for your suggestion. In our PCA analysis, elevation and topographic roughness were represented in the first dimension, while slope was in the second dimension. In our structural equation modeling analysis, we chose to represent the axis that had a greater influence.

Reviewer #2: This paper investigates forest inventory data across two surveys in 5 year increments (2016 and 2021). The authors test to see whether forest productivity across this time period is predicted/explained by diversity (which would be indicative of the complementarity effect) or by functional traits (which would be indicative of the mass-ratio hypothesis). The authors focus on multiple models examining the effect of either traits or diversity individually (along with additional predictors such as topography), then combine predictors into a single model to determine which are most predictive, and then finally utilize a path model to account for indirect effects and determine the most important predictors. They find that functional traits are better predictors of productivity in this forest while diversity is not, suggesting that the mass ratio hypothesis is applicable in this forest while the complementarity effect is not. However, they do suggest that there may still be a complementarity effect, suggesting that it is also influenced by stand density and DBH which appear positively related to productivity in their results.

Line 39: The term “mass-ration hypothesis” is used throughout and is defined as “traits of high-yielding species in a community play a major role in determining ecosystem function.” I think the authors instead mean the “mass-ratio hypothesis.” The paper they cite in the definition call it the mass-ratio hypothesis, as do other papers they cite elsewhere. The only other paper I can find that calls it “mass-ration” is also from China, which makes me suspect this may be a translation error.

Response: Thank you for your suggestion. We have revised it in the article, See lines 45.

Line 41: “Effect” should be “effort,” I think. There are a lot of similar typos and word transposition throughout the manuscript that should be corrected.

Response: Thank you for your suggestion. We have made changes in the full text

Line 42: Uncertainties about what? Be clear.

Response: Thank you for your suggestion. We have made changes in the article and see lines 48-49 for the updated text. Changed“Despite much effort in understanding the relative importance of these mechanisms, significant differences still exist among different forest types”.

Lines 31-43: The way that many of these sentences and paragraphs are constructed is somewhat disjointed. It is difficult to follow the train of logic from sentence to sentence. For example, the sentence introducing the complementarity effect seems to come out of nowhere until I read a little more, where it becomes clear that this was an intended example for the previous sentence. I would recommend reading your work out loud to yourself, to make sure that the points you are trying to make are flowing well from sentence to sentence.
Here is an example of how you could revise the beginning paragraph. This example keeps all the same points, but has better flow:
Biodiversity and ecosystem functioning have been extensively researched over the past three decades. These studies consistently show that taxonomic diversity significantly impacts forest productivity and biomass accumulation at both local and global scales. For example, the complementarity effect suggests that higher tree diversity in plant communities enhances resource-use efficiency, thereby boosting forest productivity. However, taxonomic diversity alone doesn't account for these dynamics; functional traits shaped by different species strategies are also critical for species interactions and community assembly. These traits play a pivotal role in driving ecosystem processes. The mass-ratio hypothesis, for instance, posits that the traits of dominant species largely determine ecosystem function, often quantified by the community-weighted mean of functional trait values. Despite significant advances in understanding these mechanisms, uncertainties remain about their influence across different forest types.

Response: Thank you for your suggestion. We have revised the article. For the updated version, please see lines 31-49

Line 49: Little odd to be talking about the complementarity effect here like you’re just introducing it when you also discuss it above. Also, is the niche complementarity effect different from the complementarity effect? It’s defined differently here.

Response: Thank you for your suggestion. We have revised the problem and corrected it to complementary effect in the article. See lines 35-37 for the updated text

Lines 39-82: You spend a lot of time in the coming pages talking about mass-ratio hypothesis, the complementarity effect, etc, but you really don’t spend any time expanding on those ideas in the introduction. You mention what they are, but the introduction doesn’t really provide a link between them and what you’re doing in this paper. I should be able to follow your logic all the way through to the final hypotheses, and then you tell me what you’re expecting to find based on the theories (mass ratio, complementarity) that you’re looking at.

Response: Thank you for your suggestion. We have revised the question you raised. In the introduction, we clarify the content and establish the relationship between the mass ratio effect and the complementary effect, as well as outline the objectives of this study. The primary aim of this paper is to identify which factors predominantly influence the complementary effect and mass ratio effect in mixed evergreen-deciduous broad-leaved forests within the Middle Subtropical region.

Lines 105-106: I think you should be careful when using traits from plants of the same genus to replace missing ones from your species list. For example, the Rocky Mountain Maple has a mature height of 30 feet, while the Red Maple has a mature height of ~70 feet. They are both in the genus Acer, but if you were to use data from one to replace the other then you’d end up with erroneous results.
Response: Thank you very much for the reviewers' suggestions, which have significantly expanded our understanding. We would like to provide a detailed explanation.When collecting the functional traits of the two studied plant species, we were able to retrieve data on the maximum tree height for over 97% of the species in our plots from the website https://iplant.cn, with only a few rare species not found. For these rare species, we searched within the same genus and closely related species to estimate an approximate maximum tree height.Regarding wood density, one of our students measured the wood density of over 30 dominant species in our plots. For other species, we supplemented our data using the website [http://datadryad.org/handle/10255/dryad.235](https://www.try-db.org)), where we found data for most species, although about 10 species still lacked relevant information. In such cases, we substituted missing data with traits or averages from related genera. Additionally, we compared our results with numerous previous studies and found that our findings are consistent with theirs.
Lines 111-113: These sentences are a little unclear. I think you’re trying to say that forest productivity is the sum of how much biomass for trees reaching at least 5 dbh was added per species, divided by the time interval, which is annual forest productivity? I’m not sure, though.

Response: Thank you for your suggestion. Changed to “Forest productivity was estimated as the annual biomass increment of all trees reaching at least 5 cm DBH from the first census (T0) to the last census (T1), and divided by the time interval.”

Line 116: If you are focused on the mass-ratio hypothesis, the community weighted mean is probably one of your more important variables. Here is my interpretation on why you’re using it (correct me if I’m wrong):
Community weighted means (CWMs) are central to understanding the mass ratio hypothesis because they capture the dominant traits within a community and their impact on ecosystem functioning. The mass ratio hypothesis posits that the functional traits of the most abundant or dominant species within a community drive ecosystem processes more than rare species or overall species diversity. Therefore, using CWMs helps quantify the average trait values weighted by species' abundances, reflecting the community's trait composition.
The problem here is that this is not clear from the manuscript itself. I had to read through other papers to figure this out. You need to explain why you’re using CWMs and how they link to the mass-ratio hypothesis. As the paper stands now, you have the non-weighted variables essentially front and center with the CWMs buried among other variables, and in some cases the non-weighted variables (e.g., wood density) do not have convincing linear relationships with productivity (figure 1e).
In fact, I’d go so far as to say that the increased strength of the linear relationship of CWM_WD_ in comparison to general wood density is itself really interesting, but it isn’t discussed anywhere. What does this mean for the mass-ratio hypothesis in your forest plots? Etc.

Response:Thank you for your suggestion. This issue indeed makes it difficult for us to provide a reasonable explanation. The inherent characteristics of tree functional traits indicate that "tree species with low wood density tend to grow faster and accumulate higher growth rates and productivity. Additionally, maximum tree height was significantly and positively correlated with productivity."

Line 118: The terms “stem density,” “stand density,” and “wood density” are used throughout the manuscript. In some cases, it’s hard to figure out which one you’re using. For example, wood density is provided as a trait, while stand density is a separate variable. Stem density isn’t mentioned until line 183 (results), and it’s in the same model as CWM_WD_ – so I would anticipate that if it were the same as wood density it would be collinear? But if it’s supposed to be stand density, then that is a different variable completely.

Response: Thank you for your suggestion. In the entire manuscript, we have modified the text by changing "stem density" to "stand density." Since "stand density" and "wood density" are two different variables, there is no collinearity effect.

Line 121: Why did you decide to use convexity? Not saying you shouldn’t use convexity, just would like a little more in there on why. I feel like elevation and slope are common so I don’t think you’d need to explain that in any more detail, but I’ve seen convexity relatively less often so it would be good to have your reasoning in there.

Response: Thank you for your suggestion. Changed to “We used elevation, slope, and convexity to represent terrain undulation, steepness, and curvature, providing a comprehensive assessment of topographic features”. See lines 134-135.

Line 128: Could you give a little more detail on how you standardized your variables? It would be good to know more so that a reader knows that effect sizes in your models will be impacted.

Response: Thank you for your suggestion. Before fitting subsequent models, all metrics were standardized by calculating z-scores to aid in model convergence. See lines 133-135.

Lines 128-130: If I take your writing at face value, it seems to suggest that variables with VIF less than 3 were collinear and removed from the analysis. I don’t think that’s what you mean, since larger VIFs are more indicative of multicollinearity. I assume that you did indeed remove variables with larger VIFs and it’s just a clarification issue?

Response: Thank you for your suggestion. Chaned to “To remove the multicollinear variables, we selected non-collinear with variables with a variance inflation factor (VIF) of less than 3”. See lines 142-149.

Lines 128-130: I interpret from your writing that you removed all variables that had VIF over 3. However, if there are two collinear variables and you remove one of them, the other will no longer exhibit the collinearity problem and can be kept in a model. Then you need to go into more detail on which one you kept and why, etc.

Response: Thank you for your suggestion. We considered this from two aspects: first, the variable that was deleted had a relatively small or insignificant effect on productivity; second, we removed the variable with a higher VIF.

Lines 135-137: Nice idea! This analysis type works well here.

Response: Thank you!

Lines 136-137: What was your preconceived hypothesis? You don’t need to write in the entire code for the SEM model. But you should give a basic overview of what you hypothesized should interact directly and indirectly.

Response: Thank you for your suggestion. Our hypothesis is based on several previous studies or similar research experiences. To express this more directly, we did not formulate a preconceived hypothesis. We also consulted relevant researchers who agreed with our approach, and we are confident that our results are both reliable and reasonable.

Lines 137-140: As I understand it, a given SEM variable is a number (e.g., a tree only has one number for “max tree height”, and so including it in the SEM you’ll have a collection of “max height” values for each tree). But you’ve made a PCA and selected both axes and included them both as a single variable in the SEM? I don’t know how you did that. If that is what you did and it has precedent in the literature, you should explain it. If that isn’t what you did and I’m misunderstanding it, you should then explain it in greater detail.

Response: Thank you for your suggestion. We referred to the methods outlined in the relevant literature, as seen in Rodríguez-Hernández, D.I., Deane, D.C., Wang, W., Chen, Y., Li, B., Luo, W., Chu, C., 2021. Direct effects of selection on aboveground biomass contrast with indirect structure-mediated effects of complementarity in a subtropical forest. Oecologia 196(1), 249–261.

Lines 139-142: Does this mean that the fact that you have essentially opposite effects of the two CWM traits on productivity doesn’t matter? I was wondering, because I see that you have a negative -0.54 in the SEM between the CWM traits and productivity and wasn’t sure what that meant for the two different effect sizes. If that’s the case, you should explain this in more detail, because it’s a little confusing.

Response: Thank you for your suggestion. We have carefully verified that the path coefficients are correct; it was the original Figure 3b that was not clearly expressed, and we have revised Figure 3b. The magnitude of the path coefficients indicates the strength of the effect, but does not represent the direction (positive or negative). See Figure 3b.

Line 153: As far as I can tell, you don’t report the output of the PCA analysis anywhere. That should at least be in supplementary material so that readers can see that output, with some explanation of how to read the output.

Response: Thank you for your suggestion. I have provided a more detailed description of the relevant sections in my manuscript. We initially considered this issue, and due to the limited supplementary material, we chose a simpler approach.

Lines 161-163: Figure 1d looks like a logarithmic transformation would work for maximum tree height (you don’t have to do it, just wondering if that would be a better fit). Also, while you do have significance in 1e your R^2^ value is close to 0. It really looks like you’ve got one or two influential points in the bottom right of your graph. I’m not wholly convinced you’ve got a real pattern in 1e.
Addendum: I missed this on my first pass through, but figure 1e is misleading. On figure 1c and 1d, you have p<0.001. However, on figure 1e, you have p<0.05. Standard practice is to have < only when p is so small that it’s silly to report such a small number, but to report exact p values once we start getting closer to 0.05 so readers can have full clarity of the statistics. By putting p<0.05, it hides how close to 0.05 the statistic actually is.

Response: Thank you for your suggestion. The relationship between wood density and productivity for each species reached a significant level (P < 0.05). The correlation values are r = -0.224 (*R*^2^=0.05) and P = 0.014.

Line 164: I really like how clean and well organized your figures look.

Response: Thank you for your compliment.

Lines 167-177: The paragraph discussing the correlations and statistical results presents several relationships with very low R² values, close to 0, while still implying that these relationships have substantive meaning. This can be misleading, as very low R² values indicate that the variables explain only a minimal portion of the variance in productivity, suggesting that these correlations may not be practically significant despite their statistical presentation. There are also other models that are presented as “negatively correlated” with p> 0.05 and R^2^ =0. I would not consider these to be correlated at all.
Additionally, providing context on the effect sizes for each model would be valuable in understanding the practical implications of these correlations. Without this context, the reader may be misled into overestimating the importance of these relationships.

Response: This is good advice. We have revised it. See lines 188 and 190.

Line 184: I would personally consider the Rao’s Q index as not significant (it rounds to 0.05, and even though it is technically below 0.05 I start getting a little iffy once we get p>0.03).

Response: Thank you for your suggestion. Changed to “Rao's Q index had a weak, non-significant negative effect productivity”. See line 200.

Line 187: You might want to remind people that these are standardized variables, which will impact effect size.

Response: Thank you for your suggestion. We have revised it. See lines 197.

Line 191: You say CWM had strong positive direct effects on productivity, but the effect is negative. Also, you say it’s a direct effect but the arrow in figure 3 is dashed, which in my experience represents a different kind of effect? Or at least one that wasn’t directly modeled…might be good to clarify.

Response: Thank you for your suggestion. We have carefully verified that the path coefficients are correct; it was the original Figure 3b that was not clearly expressed, and we have revised Figure 3b.

Line 197: Personal preference again: I dislike bar graphs. I think you can have the same effect with just a point graph, or even a point graph connected with lines…but again, this is my own personal preference, and I recognize other people really like bar graphs so it’s up to you.

Response: Thank you for your suggestion.This is my personal preference and I like to present my results in bar charts.

Lines 203-209: Nice summary here. Could use some cleaning up (e.g., there’s an instance where the same word is used twice in the same sentence, “increased with increasing’).

Response: Thank you for your suggestion. Changed to “species productivity increased significantly with increasing species abundance”. See lines 231.

Line 212: While maximum tree height does support your point, I think your wood density results are overstated here. Also, it would be beneficial to expand upon your reasoning behind the resource allocation explanation, providing more detailed context. For example, you could elaborate on how maximum tree height increases as trees allocate resources to structural growth, whereas faster-growing, less dense trees might prioritize rapid growth over wood density. Including additional references to support your interpretation will strengthen your argument. It might be better to discuss CWM_WD_ here to support your point, since that relationship is much more persuasive.

Response: Thank you for your suggestion.We have revised the article. Please refer to lines 238-248 for the updated text

Lines 214-215: You’re drawing conclusions regarding patterns of productivity from mostly non-significant models with incredibly low R2. 2e is the only one of these that has p < 0.01, but the R2 value is really low. I also suspect that the significance in 2e is driven by one or two influential points.

Response: Thank you for your suggestion. Changed to “Bivariate analysis indicated that three taxonomic diversity indices and functional trait diversity (Rao's Q index) showed a low correlation with forest productivity, with all statistic being at a non-significant level except for the Shannon-Wiener index”.

Lines 217-218: Why does that demonstrate that the complementarity effect is not prominent in this forest? Are there other examples of this being the case in the literature?

Response: Thank you for your suggestion. Based on Reviewer 1's suggestions, we have revised our explanations and discussions. See lines 238-248.

Line 218: You’re making a point here about the complementarity effect, then you switch to talk about mass-ratio, then you go back to continue the discussion about what your results mean for the complementarity effect in 234-244. You need to have a complete, seamless argument about the effect before you move on to mass-ratio.

Response: Thank you for your suggestion. We have made detailed revisions. See Lines 238-248.

Lines 219-220: Why?

Response: Thank you for your suggestion. Changed to “Functional trait identity plays a critical role in regulating biomass stocks in global forest metacommunities”.

Lines 222-230: You need a lot more detail on what each of these results mean for the ecosystem, for the mass-ratio hypothesis, etc.

Response: Thank you for your suggestion. We provided a more detailed explanation of the effects of these two functional traits on tree growth.See lines 269-284 for the updated text

Line 233: Why do they support the mass-ratio hypothesis? Show, don’t tell.

Response: Thank you for your suggestion. Changed to “This finding emphasizes that resource-acquisitive species with greater maximum height and lower wood density contribute more to forest productivity than resource-conservative species with lower greater maximum height and high wood density, aligning with the mass-ratio hypothesis in these natural forests.” See lines 275-279.

Line 234: What does “Complex complexity” mean? Also, it feels like you’re using the same word twice in a row, even if the meaning is different. Did you mean “canopy complexity?”

Response: Thank you for your suggestion. We have revised the article so that "complex complexity" is meant to mean "structural complexity".

Lines 242-244: Is it? You said earlier that the effect of taxonomic and functional diversity means that the complementarity effect is not prominent in this forest. Although you say in 235-236 that a variety of studies show that structural diversity has a greater impact on productivity, your SEM results shows that the comparative importance of functional traits is way more important than structural complexity. Also, why aren’t the structural complexity variables also functional traits?

Response: Thank you for your suggestion. We also found through correlation analysis that the relationship between two community-mean trait values (CWMMH and CWMWD) and productivity is stronger than that of structural variables, In Figure 2. SEM yielded similar results.

Lines 263-266: This feels contradictory.

Response: Thank you for your suggestion. We made some corrections. See lines304-310.

Lines 269-270: Which is important because?

Response: Thank you for your suggestion. We have revised the article. Please refer to lines 312-319 for the updated article.
