# Supplementary material for: Functional trait identity regulates productivity better than tree diversity and structural complexity in subtropical mixed-species forests
Source: PLoS One. 2025 May 30;20(5):e0324541. doi: 10.1371/journal.pone.0324541 (PMC12124508; doi:10.1371/journal.pone.0324541)
Supplement: Attachment — Submitted filename: Response to Reviewers 1.docx [file pone.0324541.s003.docx]

Dear Editor-in-Chief,

We express our gratitude to the Editor-in-Chief for considering our research article and providing us with the valuable opportunity to revise. We appreciate the guidance and insightful suggestions provided by the Editor-in-Chief and the reviewers, which have significantly contributed to improving the quality of our article.

We have meticulously addressed each suggestion made by the reviewers, making careful revisions throughout the entire manuscript. If any further clarification or modification is needed, please feel free to contact us. We are committed to ensuring that our article meets the standards of the journal.

Thank you for your thoughtful consideration. We look forward to hearing from you soon.

Yours sincerely,

Xiao Yu Chen

On behalf of all co-authors

Line 28 still reads RATION and not RATIO

RE: Thank you for your suggestion.We have made changes in the article and see line 29 for the updated text.

Line 33 the TreeDivNet (Public[https://treedivnet.ugent.be/publications.htmlations](https://treedivnet.ugent.be/publications.htmlations) – TreeDivNet) publishes extensively on Biodiversity and Ecosystem Function relationships please consider adding a refennce here for the list of pubblications you can find in the website. For example:
Enhancing Tree Performance Through Species Mixing: Review of a Quarter- Century of TreeDivNet Experiments Reveals Research Gaps and Practical Insights. Current Forestry Reports - [https://doi.org/10.1007/s40725-023-00208-y](https://doi.org/10.1007/s40725-023-00208-y)
Or
RE: Thank you for your suggestion. We have re-inserted the relevant references through the channels you provided, see line 34 for the updated text

Line 34 PROMOTES not PROMOTED

RE: Thank you for your suggestion. I have revised it. See Line 35.

Line38 COMPLEMENTARITY not COMPLEMENTARY

RE: Thank you for your suggestion.We have corrected similar errors throughout the article

Line 44 you state “making them more likely to have a positive impact on ecosystem functionality” while it should be “making them more likely to include at least one species with high performance in the ecosystem function of interest”

RE: Thank you for your suggestion. After reviewing a large amount of literature, we found that the common expression is "the selection effect states that more diverse communities are likely to contain more species with high performance in ecosystem functions." See lines 41-42

Lines 59 to 62: The observed positive correlation between variations in individual tree size and biomass (or productivity) may be attributed to the niche complementarity effect, which posits that forest communities exhibiting greater structural complexity can enhance resource use efficiency, thereby increasing forest functioning [6,17,19,20].
Something wrong with this sentence: it speaks of the correlation between individual tree size and productivity, which is always true in any kind of context and thus it is not clear how it can be attributed to niche complementarity. Clearly something different has to be stated in the first prat of this sentence.

RE: Thank you for your suggestion. It was indeed an incorrect expression — it should not be "individual tree size," but rather "tree-size inequality" or "tree-size variability." We have corrected it accordingly. See lines 56-59

Line 104 The largest tall and diameter Highest and largest tree attained 25.6 m and 64.8 cm in diameter, respectively.
RE: Thank you for your suggestion. I have revised it. Changed “The maximum tree height and diameter at breast height (DBH, measured at 1.3 m height) reached 25.6 m and 64.8 cm, respectively”. See Lines 104-105

Line 117: replacing missing values can be tricky: please state for which traits and species the trait was not available and with what they were replaced. This is particularly important if one of the species for which you don’t have traits is one of the seven main species. Also not that instead of using the value of the closest species you could use the average of the genus.

RE: Thank you for your suggestion. In this study, the substituted plant functional trait values constituted only a minor proportion. We compared the results obtained by using similar genus values and those obtained by using the average genus value, and found that both approaches had negligible effects on the final outcome.

Lines 142-144: something wrong here, it sounds as if you are looking for the correlation between the trait of one species and productivity which is not possible because there is no variance with one value, so it is not clear what has been done. Please clarify.
RE: Thank you for your suggestion. I have revised it. Changed “Within the 6-ha plot, we analyzed the relative contribution of different species to overall productivity.”. See Lines 144-145

Line 173: “Within a 6-ha plot, stand productivity ranged from 0.53 to 2.82 Mg yr–1.” It is not clear her and in the sentences below if the data is actually referring to the 150 plots of the experiment. So you are saying that the least productive plot has 0.53 and the most productive has 2.82 Mg yr-1? Please clarify.
The values you give for each species are higher than the highest value reported above, so perhaps you are speaking of the productivity of the species across the 6ha experiment? It might help to report the productivity per hectare.

RE: Thank you for your suggestion. Here, we present the productivity values of each species and their respective proportions at the species level, which have been appropriately adjusted.See Lines 178-179. Regarding the stand productivity value you mentioned, it ranges from 0.53 to 2.82 Mg·yr^-1^. Specifically, on the 20×20 quadrat scale, the minimum quadrat productivity value is 0.53 Mg·yr^-1^, while the maximum quadrat productivity value is 2.82 Mg·yr^-1^.See Line 192

Lines 180: here you are reporting the correlation between the productivity of a plot and the MH of the tallest tree of the CWMmh? Please clarify.

RE: Thank you for your suggestion. I have revised it. Changed “The analysis of the relationship between functional trait values and productivity of each species in this plot showed that MH was significantly and positively correlated with productivity (*R*^2^=0.22, *P*＜0.001; Figure 1d), whereas WD was negatively correlated with productivity (*R*^2^=0.05, *P*＜0.05; Figure 1e). See Lines 184-185
